# Supplementary material for: A Participatory, Needs-Based Approach to Breastfeeding Training for Confinement Centres
Source: Int J Environ Res Public Health. 2022 Sep 1;19(17):10914. doi: 10.3390/ijerph191710914 (PMC9517788; doi:10.3390/ijerph191710914)
Supplement: Supplementary file 1 [file ijerph-19-10914-s001.zip › Supplementary File S1. Questions asked during CC dialogue.pdf]

Supplementary File S1: Questions asked during the CC dialogue

1. What are you currently doing well in terms of supporting breastfeeding?
   What are barriers that you face with breastfeeding in your centre?
   If we were to put up an educational package, what would you like to be in the package?
2. Would you prefer the workshop to be on weekdays or weekends?
3. How long would you prefer the workshop to be? (half day, full day, 2 days) The group to agree on one answer
4. What would you consider a reasonable fee to pay for a full day workshop (would you expect tea and lunch)
5. How many staff will you allowed to attend at one time?
6. How many staff do you have?
7. Would it be ok to have some of the topics taught in English? How about Malay?
8. How else can we help your centre?
