# Supplementary material for: A Participatory, Needs-Based Approach to Breastfeeding Training for Confinement Centres
Source: Int J Environ Res Public Health. 2022 Sep 1;19(17):10914. doi: 10.3390/ijerph191710914 (PMC9517788; doi:10.3390/ijerph191710914)
Supplement: Supplementary file 1 [file ijerph-19-10914-s001.zip › Supplementary File S2. Self reflection form.pdf]

Supplementary File S2: Self reflection form

What can my centre do to further support breastfeeding?

**Practice**	**Yes/No**	**What can my centre do to further support breastfeeding? (highlight the ones you need someone to help you with)**
**(Step 1) Breastfeeding Policy**<br>• Does your centre have a breastfeeding policy?<br>• Is this a written policy?<br>• Are there any free or low-cost formula supplies, or any other promotional material from formula milk companies, e.g. calendars supplied by milk companies with their logos?		
**(Step 2) Staff competency in breastfeeding**<br>• Are you/your staff trained in supporting breastfeeding among the mothers? (Such as attending breastfeeding management course)<br>• If "Yes", how many of them are trained? (Percentage)		

**(Step 3) Education to mothers**<br>• Do you organise breastfeeding education to mothers at your centre?<br><br>• Do you have at least one contact with mothers (during their pregnancy) to discuss breastfeeding?<br><br>• Do you encourage women to breastfeed exclusively for 6 months?<br><br>• Do you encourage women to continue breastfeeding up to 2 years or more?<br><br>• Do you tell fathers about breastfeeding, so they know its importance and how they can help support breastfeeding?		
**(Step 4) Skin to skin contact**<br><br>• Do you encourage mothers to have skin to skin contact or kangaroo care with baby?		
**(Step 5) Support mothers in breastfeeding**<br><br>• Do you check if the mothers are breastfeeding correctly (e.g. positions, baby latching correctly, identifying a let-down reflex)?		

• Do you teach mothers how to express and store breastmilk?<br><br>• Do you encourage mothers to **avoid** giving baby other food/drinks below 6 months (e.g. cereal mixed with milk, water, glucose)?<br><br>• Do you help mothers with breastfeeding problems (such as painful nipples, breast engorgement etc)?		
**(Step 6) Supplementing with formula/water**<br><br>• Do the babies in your centre receive food or drinks other than breast milk such as infant formula, water/glucose? (Unless advised by healthcare personnel for medical reasons)		
**(Step 7) Rooming-In**<br><br>• Are mothers and babies encouraged to be together day and night?		
**(Step 8) Responsive feeding/ Breastfeeding on demand**<br>• Do you encourage mothers to feed the baby as often as the baby wants?<br>• Do you teach mothers to **avoid** 'training their babies' by giving a		

time limit for each feed? (e.g., only feed for 10 minutes then stop)<br><br>• Do you tell mothers to give the first breast until supply finishes and offer the second breast if the baby still wants to breastfeed?		
**(Step 9) Counselling mothers on the risks of using bottles and pacifiers**<br><br>• Do you help mothers avoid milk bottles and pacifiers when baby is less than 2 weeks old?		
**(Step 10) Referring mothers to breastfeeding support groups after confinement stay**<br>• Are you aware of the breastfeeding support groups in your community?<br><br>• Do you refer these mothers to breastfeeding support groups after their confinement stay?		

List 3 changes you would make in your centre after this workshop. We will follow up with you in 4 months time to see how you are progressing, and if you need our help. To enable us to contact you, kindly leave your name / centre's name and contact number.

1.

2.

3.

Name:
Centre’s name:
Contact number:
