# Supplementary material for: A Participatory, Needs-Based Approach to Breastfeeding Training for Confinement Centres
Source: Int J Environ Res Public Health. 2022 Sep 1;19(17):10914. doi: 10.3390/ijerph191710914 (PMC9517788; doi:10.3390/ijerph191710914)
Supplement: Supplementary file 1 [file ijerph-19-10914-s001.zip › Supplementary File S3. Pre and post workshop test (3 languages).pdf]

Supplementary File S3: Pre- and Post- Workshop
Test Soal Selidik Pra dan Pos Bengkel
课前课后问卷调查

Participant ID:
Nombor rujukan:
参加者编号：

1. How long should a baby be feeding on breastmilk only (exclusive breastfeeding)?
Berapa lama seseorang bayi harus minum susu ibu sahaja?
宝宝纯母乳喂养的时间应该是多长？

A.	1 month	1 bulan	1 个月
B.	3 months	3 bulan	3 个月
C.	4 months	4 bulan	4 个月
D.	6 months	6 bulan	6 个月

2. Does a breastfeeding baby need to drink additional water?
Adakah bayi yang menyusu susu ibu perlu minum air tambahan?
母乳喂养的婴儿是否需要补充水份？

A.	Yes	Ya	需要
B.	No	Tidak	不需要
C.	Don’t know	Tidak tahu	不知道

3. Which of the following indicates that the baby has had enough milk?
Di antara berikut, yang manakah menunjukkan bayi sudah menerima susu yang mencukupi?
以下哪项表明婴儿得到足够的母乳？

A.	Baby does not cry a lot	Bayi tidak menangis dengan banyak	宝宝很少哭
B.	Baby pass urine and motion	Bayi ada kencing and berak	宝宝有小便和大便
C.	Baby pass urine and motion and gain weight	Bayi ada kencing and berak serta berat bayi naik	宝宝有小便，大便和增加体重
D.	Baby pass urine and motion and sleep through the night	Bayi ada kencing and berak serta bayi tidur sepanjang malam	宝宝有小便，大便和入睡一整晚

4. Can a baby with jaundice breastfeed?
Adakah bayi yang mengalami jaundis (kuning) boleh menyusu susu ibu?
有黄疸的婴儿可以喝母乳吗？

A.	Yes	Ya	可以
B.	No	Tidak	不可以
C.	Don’t know	Tidak tahu	不知道

5. Which is the best way for a mother increase her milk supply?
Apakah cara yang paling berkesan untuk seseorang ibu menambahkan bekalan susunya?
母亲增加母乳供应的最佳方法是什么？

A.	Drink more water	Minum banyak air	喝多多水
B.	Eat healthy food	Mengamalkam pemakanan sihat	吃健康饮食
C.	Feed baby on demand including night	Menyusukan bayi mengikut kehendak bayi siang and malam	按照婴儿的需要母乳喂养，包括晚上及半夜时
D.	Take medicine	Mengambil ubat	服药

6. When should we wash our hands?
Bilakah seseorang harus mencuci tangan?
我们在什么时候应该洗手？

A.	Before and after holding a baby	Sebelum and selepas memegang bayi	抱婴儿之前和之后
B.	After touching baby's cot	Selepas menyentuh katil bayi	触摸婴儿的床后
C.	After changing baby's diaper	Selepas menukar kain lampin bayi	换婴儿的尿片之后
D.	All of the above	Kesemua di atas	以上所有的选择

7. When a mother has nipple pain during breastfeeding, she should
Apabila seseorang ibu mengalami kesakitan pada putting payudara, dia harus
当母亲在母乳喂养期间出现乳头疼痛时，她应该

A.	Stop breastfeeding	berhenti penyusuan susu ibu	停止母乳喂养
B.	Apply oil on her breast	membubuh minyak ke atas payudara	在她的乳房上涂油
C.	Express milk and feed using bottle	mengepam susu and memberikan susu dengan botol	挤奶然后用奶瓶喂母乳
D.	Check if baby is attached correctly to her breast	periksa sama ada bayi melekap dengan betul pada payudara	检查婴儿母乳喂养的姿势是否正确

8. Which of the following will cause a mother to have poor milk supply?
Di antara berikut, yang manakah menyebabkan seseorang ibu mengalami kurang bekalan susu?
以下哪项会导致母亲供应母乳不足？

A.	Small breasts	Payudara kecil	母亲的乳房小
B.	Preterm baby	Bayi tidak cukup bulan	早产的婴儿
C.	Caesarean section	Pembedahan Cesarian	剖腹产
D.	Adding formula feed	Tambahan susu formula	添加奶粉
