# Supplementary material for: A Participatory, Needs-Based Approach to Breastfeeding Training for Confinement Centres
Source: Int J Environ Res Public Health. 2022 Sep 1;19(17):10914. doi: 10.3390/ijerph191710914 (PMC9517788; doi:10.3390/ijerph191710914)
Supplement: Supplementary file 1 [file ijerph-19-10914-s001.zip › Supplementary File S4. Participant Feedback Form.pdf]

Supplementary File S4: Essential Breastfeeding Skills Workshop for confinement care personnel

We would love to hear your comments about our workshop.

1. What do you think about this workshop?

Very bad									Excellent
1	2	3	4	5	6	7	8	9	10

2. Would you recommend this workshop to a friend?

Not Likely									Highly Likely
1	2	3	4	5	6	7	8	9	10

3. What you liked about the workshop

___

___

___

4. What you did not like about the workshop

___

___

___

5. Other comments:

___

___

___

___

Thank you

陪月人员哺乳培训课程

参与者反馈表

我们真盼望您给予此工作坊的宝贵意见

1。您对此工作坊有什么看法？

非常不满意 非常好

1 2 3 4 5 6 7 8 9 10

2。 您会向朋友推荐此工作坊吗？

不会 肯定

1 2 3 4 5 6 7 8 9 10

3。 您喜欢此工作坊的哪些内容？

____

____

____

4。 您觉得此工作坊有哪些需要改进的地方？

____

____

____

5。 其他意见

____

____

____

谢谢
