# Supplementary material for: A Participatory, Needs-Based Approach to Breastfeeding Training for Confinement Centres
Source: Int J Environ Res Public Health. 2022 Sep 1;19(17):10914. doi: 10.3390/ijerph191710914 (PMC9517788; doi:10.3390/ijerph191710914)
Supplement: Supplementary file 1 [file ijerph-19-10914-s001.zip › Supplementary File S5. Workshop programe.pdf]

Supplementary File S5: Programme

Essential breastfeeding skills workshop for confinement care personnel

Time	Programme
0800	Registration
0830	Ice breaking activity
0845	Breastfeeding – it's more than just making milk
0915	How does milk get to baby?
0945	How to talk to mothers about breastfeeding
1030	Break
1100	Is there a right way or wrong way to breastfeed?
1130	Is mummy breastfeeding correctly?
1200	How do we know baby is getting enough milk?
1230	Steps to successful breastfeeding
1300	Lunch
1345	Making breast models & group photo
1400	How to help mothers express milk
1445	How to handle common breastfeeding problems
1545	Making breastfeeding work and help your center excel
1700	Closing
